# Supplementary material for: Are carbon emissions trading and green financial instruments synergistic? -Comprehensive quantitative research based on content analysis
Source: PLoS One. 2024 Mar 7;19(3):e0298601. doi: 10.1371/journal.pone.0298601 (PMC10919601; doi:10.1371/journal.pone.0298601)
Supplement: S1 Data — (ZIP) [file pone.0298601.s001.zip › Data/地级市Z_F工作报告.zip!/║■▒▒/╨ó╕╨╩╨/└┤╫╘╣1⁄2╓┌║┼í╛┬φ┐╦╩2╛▌═°í┐_2019.docx]

各位代表：

现在，我代表市人民政府向大会报告工作，请予审议，并请各位政协委员和列席人员提出意见。

一、2018年工作回顾

2018年，是极不平凡的一年。习近平总书记视察湖北。市委六届六次全会胜利召开。孝感的发展，站在新起点。一年来，我们坚持以习近平新时代中国特色社会主义思想为指导，在省委、省政府和市委坚强领导下，紧紧依靠全市人民，落实“十抓十促”[1]路径，真抓实干、埋头苦干，较好完成了市六届人大三次会议确定的目标任务（见附件一）。

经济运行稳中有进。预计地区生产总值增长7.8%左右，规上工业增加值增长8%以上，固定资产投资增长11%以上，社会消费品零售总额增长11%以上，地方一般公共预算收入可比增长11.3%。主要经济指标增速一举扭转多年下滑趋势，达到或超过全省平均水平。

经济质效明显提升。引进过百亿元项目6个。入库、在建、纳统项目1028个。新增科技型企业146家，申报高新技术企业138家，纳税过亿元企业达15家，孝南纸品晋级百亿产业集群。税收占一般公共预算收入的比重提高9.8个百分点。服务业增加值占比提高0.5个百分点。万元GDP能耗完成省定目标，亿元GDP地耗率下降5.39%。

攻坚之战初战告捷。全国驻村帮扶工作座谈会在孝感召开，孝昌顺利通过国务院脱贫攻坚督查，全市脱贫59457人，出列77个村。孝感城区空气质量明显改善，8个国考断面水质全部达标，工业园区污水处理设施全部建成。社会风险防控有力，“扫黑除恶”居全省第一方阵。依法规范举债融资，清收、处置不良贷款23.24亿元，守住了不发生系统性、区域性风险底线。

发展平台更加坚实。孝感与圣彼得堡赤卫军区达成国际友城意向，46家企业在首届进博会上开展国际合作，89家企业在广交会上收获2亿美元订单，全市沿“一带一路”[2]出口3亿美元，红贡茶出口俄罗斯和中东欧，云梦皮草获批国家外贸转型升级示范基地。孝感通过国家园林城市复查，获评省级森林城市。国家农业科技园区正式批建，全市新增3个国家星创天地、2个国家企业技术中心①。

发展秩序全面优化。统一房屋征迁与补偿标准、工作流程、安置办法和执行尺度，市高新区、临空区和孝南区当年征拆房屋4778户、100多万平方米，拆迁规模超过前十年之和。整治、查处围标串标案件，联合惩戒20家失信企业，依法打击9人，判刑3人。规范砂石市场秩序，国有控股矿业公司正式运营。国土资源节约集约模范市创建深入开展，消化批而未供土地1.31万亩，处置闲置土地48宗4366亩，盘活闲置厂房34.68万平方米，矿山治理17处 291亩。开办企业只需4个工作日，备案类项目从注册到施工只需40个工作日，“多证合一”发照2.7万份，“双随机一公开”抽检市场主体25.1万次。

发展成果普惠共享。农村常住居民人均可支配收入快于经济增速。城镇新增就业65120人，困难人员就业7522人。基础养老金提标惠及70多万人，“五项社保”参保830万人次，省内异地就医即时结算。改造农村危房5110户，建成棚改房5937套，分配公租房22627套。向21.8万名困难对象救助10.3亿元，孤儿养育标准提高30%，筹资2亿元资助困难学生15万人次。对437名0-6岁残疾儿童抢救性康复治疗，“两项补贴”[3]惠及9.6万名残疾人，为51万名老年人购买意外伤害保险，高龄津贴惠及12.46万名老年人。

一年来，我们主要做了以下工作：

（一）坚持“项目为王”，“两资一促”稳增长。“三招三换”[4]成为主旋律。全面实施“一号工程”，把握产业导向和“六度原则”[5]，聘请招商顾问20名，授予荣誉市民2人，与16家中省国企战略合作。全市引进过亿元项目226个，注册178个、开工175个、投产58个，实际利用外资3.41亿美元。日商产业园新落户项目7个，聚集日资企业25家。立项争资成为主基调。研透政策方向，努力让项目入笼子、进盘子。动态策划项目629个，投资超万亿元；到位竞争性资金68.3亿元，新批重大事项和改革试点60个。争取水利投资14.1亿元，全省“补短板”工作现场会在孝感召开。争取电网投资11.22亿元，在全省率先完成新一轮农网改造。项目建设成为主舞台。专班推进，精准服务，三次集中开工项目197个，全市开工亿元以上项目252个、投资超千亿元。完成交通投资59亿元。工业和技改投资分别增长15%、22.1%。省级三类项目和市级113个重点项目超额完成年度计划，孝感城区164个市政工程完成投资98.5亿元。

（二）坚持优化服务，扶持企业促转型。加强机制建设，实现项目“三库”接续，数据“三网”[6]支撑，企业量增质优。工业经济步伐稳健。真金白银支持工业，真心实意服务企业，动态完善“六本台账”[7]，招工5.13万人次，减免税费24.1亿元，81家企业直供电降成本3500万元。全市新增 “新三板”、四板企业44家。新登记企业9891家，新增规上工业企业47家、院士工作站10家，授权发明专利300件，新增“隐形冠军”14家。评选服务标兵和技术能手99人，表彰创业示范基地9家、创业明星12人。孝南纸业、汉川童车获批省级产品质量提升示范区。服务业扩面增效。建立服务业“新进规”培育机制，持续优化产业结构。18个行业“个转企”645家，新增服务业规上企业94家，净增限上商贸单位108家。快递下乡覆盖40%的村组，快递总量和收入全省第四，大悟、孝昌获评国家电子商务进农村综合示范县。农业基础更为坚实。土地确权登记颁证基本完成，流转土地150万亩，建成高标准农田39.9万亩，受旱之年粮食总产48.55亿斤、增产0.27亿斤。新建虾稻、渔稻共作面积6.6万亩，新增农业龙头企业12家、农民专业合作社1008家、家庭农场214家，农产品加工业产值突破1500亿元。孝感香米入选省农产品区域公共品牌二十强。全国农业绿色高质高效整市制创建试点全面推进，安陆获批全国主要农作物生产全程机械化示范县，汉川南河乡入选全国农业产业强镇示范乡镇②。农旅养互促共赢。建成“四好农村路”680公里，创成农旅养融合示范点11个、星级农家乐55家，新建农业休闲、采摘园100个，两镇入选第二批省特色小镇③，安陆上榜2018“中国田园美邑200佳” ，金卉庄园获批4A级景区，应城楚珍园、龙池山庄入选全国森林康养基地，汉川天屿湖休闲社区开园迎客。全市接待游客2600万人次，旅游综合收入172亿元，分别增长13%和15%。

（三）坚持问题导向，创新供给解难题。针对实体经济困难、支柱产业不强的问题，密集出台苗木花卉、工业转型、创业投资、道路货运、旅游发展、优化营商环境、金融支持实体经济、促进民营经济和新经济发展等激励政策，为1830家企业融资、担保、续贷周转100多亿元，4家企业获评湖北民营企业100强④，建筑业产值达450亿元，新注册货运物流市场主体1743家，云图控股成为全国最大品种盐基地。针对新老城区不协调、城市“补丁”较多的问题，完成孝感城市“双修”专项规划，把40平方公里老城区划分为17个片区，分片分批改造；交通大道、孝三路和4个路口改造升级⑤；八部门、五渠道⑥联合管控、综合执法，制止违建185起，依法拆违752处，控违拆违面积超过前三年之和；孝感旧城改造第一批历史遗留问题整改完毕，第二批进入整改程序；4.5平方公里的老澴河综合治理全面启动，9个安置点226万平方米还建房全部动工。针对发展活力不强、环境不优的问题，以改革促创新、激活力、优环境，17项国家级、23项省级改革试点和75项市级改革事项有序推进，税收征管体制改革平稳接续，澴川国投转型为国有经营实体，市民“随手拍”参与智慧城管，重大项目容缺受理、并联审批、先建后验，“一张网”提前7个月实现五级联通，“互联网+放管服”改革走在全省前列。针对工作抓手不实、责任不清、结果与初衷出现偏离的问题，全面实施“五化”[8]管理，入库项目536个，涵盖74家单位的重点工程、重点工作；梳理民生“双保”清单108项；“大统计”格局基本形成，通过依法采集数据，促进项目落地，倒逼责任落实。

（四）坚持统筹协调，区域融合拓空间。汉孝一体加快融入。“五个同标准对接”[9]全面启动，公交、旅游一卡通用，医疗、疾控联防联动，跨区域护水治水深度合作。武汉城市圈环线高速孝感南段、汉孝大道相继通车，武大高速、硚孝高速动工建设，2条铁路、4条高速、3条旅游线、5条等级公路快速对接⑦。面向武汉招商50多场次，签约项目101个，引资1930亿元。三区形象不断提升。市高新区新建工业项目35个，新能源大中型客车实现年底总装下线，军民融合基地产值达135亿元。市临空区引进项目16个，3个百亿元项目奠基开工⑧。市双峰山忠园、义园竣工，景区通过省级旅游度假区验收。孝感城区增绿补绿、拆墙透绿13处，复兴大道建成通车，董永北路立交桥开工建设。城乡一体协同发展。市民下乡1.1万人，能人回乡1.4万人，兴乡企业1400多家。建设美丽乡村158个，6个村入选全省美丽乡村示范典型， 8个村镇获评全国一村一品示范村镇，5个乡镇跻身全国综合实力千强镇⑨。孝南引进华夏幸福基业共建汉孝临空产业园。汉川荣获四个全国百强称号⑩。应城获评国家知识产权强县优秀试点。5个县(市、区)纳入汉江生态经济带 ⑪，大悟纳入淮河生态经济带。

（五）坚持生态优先，呵护绿色优环境。用环境治理留住绿水青山，用绿色发展赢得金山银山，大气和水质明显改观，生态环境持续改善。环境突出问题全面整治。中央环保督察年度整改任务全部交账，督察“回头看”任务圆满完成。省环保督察178个交办事项全部办毕并公示。淘汰燃煤锅炉546台，更新改造地下油罐235个。“两禁三治一限”[10]加强扬尘管控和大气污染防治，秸秆禁烧实行网格化管理，孝感城区PM10、PM2.5平均浓度分别下降13.9%和10.6%。河（湖库）长制和路长制全面推行。把长江大保护作为政治任务，十大标志性战役、14个专项行动推进迅速。实行最严格水资源管理，饮用水源地环境问题全部整改到位，市直3处黑臭水体⑫ 整治完成。一河一策、四级齐抓、常态巡查，全市河道无非法采砂船舶。江河湖库拆围2.32万亩，水质稳定向好。明确市县乡村四级路长1521名，路长制全面实施。生态建设全面加强。风电、光伏装机118.75万千瓦，全省第二。孝感城区污水处理厂提标改造，邓家河污水处理厂投入运营,表面处理生态产业园动工兴建。占地1300亩、投资18.9亿元的静脉产业园,实质动工、加快建设。建成乡镇污水处理厂79座，配套管网1100公里。8座垃圾处理场、120座垃圾中转站覆盖城乡。关闭或搬迁养殖场955家。全市新改建“六类厕所”[11]19.41万座，云梦县“厕所革命”一年完成三年任务。精准灭荒7.16万亩，朱湖国家湿地公园获批，新增省级湿地公园2处、森林城镇3个⑬、绿色乡村159个。安陆获批国家农业面源污染治理试点。

（六）坚持改善民生，共建共享增福祉。兜底线、保基本，在发展中不断提高保障和改善民生水平。攻坚拔寨促脱贫。调实调优驻村工作队1604个，30585名干部结对帮扶11.38万贫困户。统筹资金用于精准扶贫30.98亿元，发放扶贫小额信贷5.27亿元，培植“扶贫车间”等经营主体4931个，易地搬迁13117户27343人，贫困对象就医一票制、一站式结算。表彰“脱贫光荣示范户”20户、社会帮扶先进典型10人。加大投入促公平。财政投入保基本，社会投入增供给。建成示范留守儿童之家116个，新改扩建农村福利院10所。新增公积金实缴人数1.2万人、资金25亿元。投入2.6亿元全面改薄标准化学校255处，孝感城区改扩建学校12所，义务教育划片就学、阳光招生；公开招聘教师1219人，授予“市级中小学教师专业荣誉”1980人。组建医共体10个，定向招录、公费培训全科助理医生124名，常住人口、重点对象家庭医生签约率43.7%和71.5%，免费婚检、优生孕检6.5万人次，免费筛查“两癌”2.28万人。中华孝文化高峰论坛和老博会、省楚剧艺术节、民间文艺大赛、龙舟邀请赛等活动相继举办，在省十五运会上获得金牌39枚，4人入选中国好人榜，首次评选市文明家庭50户，三地上榜省民间文化艺术之乡，两地获评全省首届特色文化村 ⑭ ，孝感连续五届荣获全国无偿献血先进单位。共建共治促和谐。网上网下一体化管控，矛盾隐患源头排查化解，打击电诈、禁赌扫黄、利剑斩毒、交通秩序整治持久深入，现发命案全破。阳光信访畅通群众诉求，分类化解处置信访积案557件。安全生产和食品安全形势向好，社会大局稳定。2143个村、286个社区换届选举。14个社区获评全省减灾示范社区，2个村（社区）获评第七批全国民主法治示范村⑮ ，应城连续三次获评全国法治县市创建先进单位。

（七）坚持党的领导，依法履职转政风。把党的领导贯穿政府工作始终，践行“十个带头”，立担当之德、树奋斗之标、行求真之为，与时俱进加强政府自身建设。提请市人大立法2部。认真执行市人大各项决议决定，自觉接受政协民主监督和社会舆论监督，办理人大代表建议180件、政协委员提案201件；政府作出重大决策、制定规范文件，先调研论证、法制审查，再集体讨论决定；行政复议102件，行政负责人出庭应诉率达86.6%，法治政府全面推进。持之以恒纠“四风”、转作风，注重角色转换、团结协作、补台补位，重点工作实行路线图管理、在线督办。加强风险内控，出台政府投资项目招投标、工程总承包、造价等系列管理办法，把牢工程质量、资金安全、廉洁行政关口，公共资金、公共资源、国有资产跟踪审计，清廉政府取得实效。

与此同时，密切联系工会、共青团、妇联，国防动员、双拥共建和优抚安置工作不断加强，人防、保密、气象、水文、档案、史志、物价、保险、慈善、外宣、通信、广播电视、支疆援恩、外事侨务、台湾事务、民族宗教、驻外和机关管理等各项工作取得新成绩。

各位代表，过去的一年，我们栉风沐雨、砥砺前行，高质量发展迈出坚实步伐，每一点成绩都凝聚着大家的智慧和汗水。在此，我代表市政府，向全市人民，向人大代表、政协委员和社会各界，向高校、驻孝部队和公安干警，向所有投资者、创业者、建设者和关心、支持孝感建设发展的各界人士、各位朋友，表示崇高敬意和衷心感谢！

安不忘危，稳不忘忧。我们清醒看到，发展不平衡不充分问题依然存在，主要是：县域之间、城乡之间、企业之间分化明显；传统产业增速放缓，新经济、新产业处于培育期，稳的基础还不牢固；实体经济比较困难，小微企业融资难、结构性用工难问题较为突出；资金、土地、环境等要素约束趋紧，招商项目落地不快，民生欠账较多，发展改革稳定面临诸多两难抉择。少数干部担当不够、作风不实、效率不高，经济发展的“懒人”思维、履职尽责的“痕迹”思维、脱离实际的“豪华”思维、不下“深水”的“官僚”思维、一团和气的“好人”思维、面对风险隐患不怒不急的“麻木”思维，不同程度存在。对此，我们将保持清醒头脑，直面现实问题，采取有力措施，认真予以解决。

二、2019年工作安排

今年是新中国成立70周年，是实现“十三五”规划的关键一年，更是决胜全面小康、实现脱贫摘帽的攻坚之年。做好今年的政府工作十分重要。总体要求是：以习近平新时代中国特色社会主义思想为指导，全面贯彻党的十九大和中央经济工作会议精神，深入贯彻落实习近平总书记视察湖北重要讲话精神，按照全省经济工作会议统一部署，坚持稳中求进总基调，以建设全省区域性增长极为目标，以供给侧结构性改革为主线，以改革开放创新为动力，全面落实“六稳”[12]工作部署，精准对接全省“一芯两带三区”[13]区域产业布局，深入实施“十抓十促”发展路径，打好“三大攻坚战”，彰显“四个融合”发展特色，提升民生保障水平，统筹推进“稳促调惠防”各项工作，奋力谱写新时代孝感高质量发展新篇章，为全面建成小康社会收官打下决定性基础，以优异成绩庆祝新中国成立70周年。

根据这一要求，我们把思路稳、目标稳、措施稳，作为“六稳”的具体行动，坚定“两保持一优先”[14]目标，聚焦“八双”实务，细化实化全年各项工作任务（见附件二），重点抓好八个方面工作。

（一）聚焦投资质效，深化项目“双增”。项目增量增质、投资增速增效，是高质量发展的要求和基座。我们以“两资一促”为载体，广泛开展项目储备提质年、社会投资促进年、项目建设竞进年活动，抓招商、争资金，备要素、建项目，全年投资增长10.5%以上。

精准策划储备项目。盘清资源家底、产能家底，完善招商指南和选商清单，带着项目找投资，提高招商针对性和精准度，做实招商选资库；坚持“三招三换”，围绕特色优势资源，聚焦六大主导产业[15]，深化央企省企合作，团队招商、顾问推介、园区引领、能人回归，引进一批建链补链强链项目，做大招商签约库；全年引进过亿元项目200个以上，利用外资增长8%。把握投资方向，紧盯政策窗口，围绕长江经济带、长江中游城市群、汉江生态经济带、武汉城市圈两型社会、大别山革命老区五大平台，突出产业转型、基础设施、社会事业、生态环保、乡村振兴五大领域，动态策划包装项目，市本级安排2000万元支持项目前期工作，滚动充实项目储备库，项目储备总额达上年投资的2—3倍，转化率30%以上；争取上级资金100亿元以上。

精细提供要素保障。开发区、工业园区、产业功能区，推行“多评合一”[16]，统一开展报建审批区域性评价。全面落实鼓励民间投资优惠政策，提振投资信心，激发投资活力，民间投资高于全市投资5个百分点。统一配齐项目秘书，全程代办手续，跟踪协调问题。重大工业项目和市政项目，多规合一、多证合一、多验合一，审批事项一单清、报批材料模板化，中介服务政府买单、业主“零付费”；完善项目在线审批监管平台，鼓励网上申报、网上审批，新开办企业限时3个工作日，工业项目38个工作日内取得施工许可证。综合运用增减挂钩、增存挂钩、迁村腾地、市域统筹、结对协作等办法对内挖潜，用足用活政策，及时报批用地指标，节约集约与供需平衡并重，及时提供用地保障。定期开展政银对接活动，与省再担保集团、资产管理公司深度合作，多渠道提供资金保障。提前备好水、电、路、气、用材、通讯等要件，全方位提供开工保障。

精心服务项目建设。强化领导领衔、专班推进，集中开工、现场拉练，“五化”管理、跟踪督办等项目推进机制，坚持“六有”[17]标准评价、检查、验收项目，实行台账管理、跟踪问效，新开工10亿元项目15个以上，过亿元在建、纳统项目110个以上。深化“千名干部进千企”活动，帮助实体经济纾困解难、增资扩能，工业投资快于全市投资2—8个百分点。服务汉十高铁建成通车，续建新建一二级公路32条，完成交通投资90亿元以上；建设水利、电网项目57个，完成投资27.8亿元。

（二）聚焦融合发展，深化城乡“双融”。城乡资本融合、产业融合，是高质量发展的必由之路。我们要顺应大势，深化四个融合，扶企业、优产业，培植新增长点。

推进汉孝融合新突破。建立定期交流机制，拟定年度合作清单，市县、部门、企业、高校、社团常态化对接合作。加快构建汉孝1小时交通圈，续建武汉城市圈环线孝感北段、硚孝高速二期、武大郑高速大悟段，动工建设孝汉应高速，全方位服务武西直通线，密切跟踪沿江高铁和3条轨道交通工作动态。市区一体、政企合作建设汉孝临空产业园，启动汉川刘隔工业园扩规，建设汉宜高速北河工业园，各开发区工业园全面承接武汉产业转移。开展农产品供应、健康养老、社会事业等全方位合作共享，鼓励A级景区、星级农家乐纳入旅游“一卡通”，促进汉孝两地景区互动。

建设军民融合新高地。提升三江航天、航天重工装备产业园，加快华中光电、大悟武船产业园建设，拓展军民结合示范基地。定期组织军工与民企对接，推动军转民、民参军，加快新能源汽车、智能装备、激光器、机场搬迁等重点项目进度，争创国家军民融合创新示范区。推动三江万山投资主体多元化改革，大洋新动力EV电机和氢燃料电池实现量产，力促新能源客车、物流车批量产销，年产值10亿元以上。深入开展双拥共建、国防教育，做好退役军人事务工作。

打造城乡融合新引擎。贯彻落实乡村振兴战略，以农旅养融合为抓手，推进村庄环境整治、公共服务、农旅养三年行动方案，深入开展“三乡工程”，探索建设共享民居、共享田园、共享农庄，吸引社会资本参与“农业+”“+旅游”“+养老”，在美丽乡村中融入旅游、养老元素，加快建设华中生态康养型旅游目的地。全盘谋划、串珠成链，以路长制为抓手，加快11条农旅养融合示范线建设，配套旅游驿站、旅游厕所、观景平台、慢行设施、旅游标牌，加快通道景观绿化，让示范线成为风景线。推进文旅融合，整合楚文化、孝文化、红色文化、李白文化、民俗文化资源，“一县一特”打造7条文旅融合带，建成一批文旅融合地标景点景区，扩大中华孝文化论坛影响力。提升“吃住行游购娱”标准，契合“商养学闲情奇”趋势，新增A级景区5家以上，创建农旅养融合示范点10个、旅游名镇名村2个、星级农家乐50家，旅游综合收入增长15%。

构建市域融合新格局。依法依规理顺市区体制机制，明晰市区财权事权和基本公共服务支出责任，规范市区综合执法机构和队伍建设，正确处理“政治领导、平等市场主体、属地管理”三者关系，发挥市直三区、孝南区多个主体积极性，共同构筑市区龙头优势。市高新区加快东区基础设施配套和主导产业提升，全力打造高新产业聚集区；市临空区全力加快产业项目建设，尽快建成汉孝同城化核心区；市双峰山全面提升旅游设施、开发度假产品、打造业态品牌，完成双峰山至木兰景区群旅游公路改扩建，创建国家级旅游度假区。支持汉川在全国百强中争先进位，支持孝南、应城冲刺全省20强；支持云梦、安陆特色发展、错位发展，抢占全省新的增长点；支持大悟、孝昌加快建设山区经济强县。

（三）聚焦改革创新，深化动能“双培”。传统动能不强、新动能成长慢，是孝感高质量发展面临的最大现实。我们坚持供给侧结构性改革主线，培优旧动能、培强新动能，增强发展动力与活力。

深化改革促开放。按时完成市县机构改革，扎实推进生产经营类事业单位脱钩改制、环保体制垂直管理、医药卫生体制、社会保障制度、商事制度等各项改革。深化农村土地制度、集体产权制度和国有农场改革。抢抓全方位对外开放和形成强大国内市场的机遇，主动融入“一带一路”和湖北自贸区建设，抓好长江经济带、汉江生态经济带、大别山革命老区振兴发展等重大战略落地，加强与国际友城交流合作，创建日商国际合作产业园，支持国家级皮草、省级童车、禽蛋和特色农产品出口基地建设，进出口总额增长6%以上，新增实绩出口企业20家。

双轮驱动促振兴。推进“四化同步”示范乡镇、小城镇综合改革、经济发达镇行政管理改革试点示范，实施特色小镇三年行动计划，积极创建国家、省级特色小镇。全面实行零门槛落户政策，城镇化率提高1—2个百分点。推进产业向城镇、园区集中，壮大七大重点成长型产业集群[18]，打造全省特色产业增长极。加快发展现代农业，延伸农业产业链、价值链，农产品加工业产值增长8%。推进国家优质粮食工程，绿色高质高效整市制创建面积200万亩以上，建设高标准农田59万亩，耕种收综合机械化率76.5%，粮食稳产在45亿斤左右。农村安全饮水巩固提升24万人。建设“四好农村路”1000公里，改造危桥80座，全面完成公路安防“455”工程⑯任务，压实路长责任，打造“安全、通达、净美”的交通环境。开展“一村多名大学生计划”和文明村镇创建活动，培训300名农村实用人才，改造5226间农村存量危房，培育中心村、保护特色村、整治空心村，弘扬文明乡风、良好家风、淳厚民风，建设100个美丽乡村、1000个农村“三小园”[19]。

鼓励创新促转型。积极申报国家知识产权示范城市，推进国家创新型城市、国家农业科技园区、产业技术研究院建设，争创一批国家和省级实验室，以创建促创新。建立市级创业投资引导基金，完善科技企业孵化器、众创空间优惠政策，实施创业投资专项奖励，深化“立业孝感”计划和技能人才振兴计划，新增高技能人才500人，新建各类孵化载体5家，转化科技成果100项以上，高新技术产业增加值增长12%。构建亲清新型政商关系，真正把民营企业和企业家当自己人，积极化解民营企业困难，表彰一批“爱国敬业、守法经营、创业创新、回报社会”的企业家，鼓励企业做强做优；创新机制盘活存量，推进“千企千亿技改”“千企上云”工程[20]，促进“两化”融合，推动制造业高质量发展，新增规上工业企业45家、高新技术企业100家、“两化”融合示范企业2家、隐形冠军6家。发展装配式、绿色节能建筑，做大做强现代建筑业。实施质量强市、品牌强市战略，弘扬“工匠精神”，培育“荆楚优品”，新增驰名商标2—5件。大力培育共享经济、数字经济，发展现代服务业，加快建设6个物流园，新增服务业规上企业50家、限上商贸单位80家。创新金融产品，新增贷款100亿元，新增上市金种子企业1家、“新三板”和四板企业30家以上。

（四）聚焦品位提升，深化城市“双修”。城市是高质量发展的平台和载体。我们全面开展生态修复、城市修补，实施城市建设绿色发展三年行动，提高颜值、提升品质，让城市生活美起来。

精细规划建设管理。统筹新老城区、地上地下空间、“三区三线”⑰管理，转化第三次国土调查成果，推进“多规合一”，全面提升规划引领水平。围绕功能补短板，加快东城区学校、医院、集贸市场等公共服务设施建设，孝感城区和市直三区新建续建市政项目196个，完成投资220亿元以上。加快建设107和316国道改扩建、董永北路立交桥、长兴三路西延伸、宝成路南延伸、槐荫大道东延伸、陈天大道对接等骨干路网，打通干线、闭合环线，确保城市外环2020年底建成通车；新建孝感城区100万头生猪屠宰厂，确保年底正式投产；联通八一水厂和第二水厂，加快建设静脉产业园5个子项目⑱。深化“城管革命”，孝感城区开展“净、绿、畅、亮、美”专项治理，申报国家卫生城市，深化文明城市和生态示范城市创建；汉川、应城、安陆开展垃圾分类试点。完善融媒体平台和智慧城管平台，推进智慧城市建设。

再造功能环境基层治理。建设新城区，不忘老城区。坚持“整体规划、分步实施，东西衔接、协调发展”原则，启动孝感老城区2个片区“双修”改造⑲，建设城站路2.2公里平战结合人防工程，提升10条干道和8个重点区域道路，亮化14条街道；加快建设春尚小区、云湖社区还建房，改造10个“三无”老旧小区，积极稳妥开展老旧住宅加装电梯工作。全面解决孝感旧城改造第二批遗留问题。老澴河综合治理完成投资66.45亿元，全面开展岸线截污、生态修复、道路和绿化配套，建成安置房12981套。落实《孝感市住宅小区物业管理条例》，加强小区综合管理，评选表彰一批优秀物业公司、和谐示范小区；在孝感主城区每个街道和6个县市城区，开展示范社区创建活动，不断推进“自治、法治、德治 ”相结合的基层治理体系建设。

传承水脉绿脉文脉。启动澴东“四河三湖”[21]水系连通，综合治理孝感城区12条内河、沟渠水体，建成区基本消除黑臭水体；完成孝感城区污水处理厂提标改造，建成市临空区污水处理厂，配套建设污水收集管网，城区污水全部达标排放，污泥无害化处理率85%以上；实施后湖渠片区雨污分流一期工程，建设城西泵站、王家漖泵站。提升城市园林绿化景观，完善槐荫公园、董永公园、后湖公园的配套设施，再建一批街头公园，改造环城通道出口绿化景观，强力推进国家森林城市创建，6个县市创建省级森林城市，应城、云梦确保创建成功。结合老澴河改造、城区“双修”划定历史街区，启动重建文昌阁，修复西湖桥，打造1.5平方公里老城印象，产城景融合塑造城市“会客厅”，加快建设中华孝文化名城。

（五）聚焦美好向往，深化民生“双保”。满足人民美好生活需要，是高质量发展的目的和归宿。我们建立健全基本公共服务标准体系，保刚性政策和上级决策部署落实、保民生承诺兑现，让人民群众共享发展成果。

实施居民增收计划。坚持就业优先，网上网下拓展就业服务，多渠道开发就业岗位，以更充分、高质量就业促居民增收。深化返乡创业三年行动，技能培训贫困家庭子女、农民工8000人次，城镇新增就业4.8万人，吸纳大学生就业创业5000人，困难人员再就业6000人，城镇登记失业率控制在4.5%以内。完善工资收入分配制度，调整并落实最低工资标准，推进公立医院、国企薪酬制度改革。抓好农民工工资治欠保支，规范农村土地、住宅有序流转，培育一批家庭农场、农民专业合作社、种养大户等新型经营主体，促进农民增收。

实施全民保障计划。完善城镇职工和城乡居民医保制度，落实企业职工养老保险基金中央调剂制度，推进工伤保险降费扩面，鼓励全民参保，“五项社保”参保835万人次。改扩建5所农村福利院，健全完善农村留守关爱服务体系，建设智慧养老服务平台，推动城乡社区居家养老。健全完善“救急难”制度，动态调整城乡低保、农村特困供养、孤儿生活养育标准，织密兜牢民生网底。棚户区改造5947套，以“新市民”为重点公积金扩面1万人。坚持“房住不炒”、因城施策、分类调控，保持房地产业稳健发展。

实施教育均衡计划。加强现代学校制度建设，提升师德师风。深入开展学前教育三年行动，统筹推进两类学校[22]建设和城乡义务教育一体化，完成217所学校全面改薄任务，开工建设市澴川学校和黄香路小学，支持社会力量办学，增加学位供给，加快消除“大班额”；探索“互联网+”远程教学，均衡城乡教育资源，深化教育教学改革，提升中考合格率和高考上线率。开展校园足球试点学校建设和中小学研学旅活动。支持市特校提档升级。推进高中阶段教育普及攻坚计划。产教融合开展职业教育，整合资源建设湖北工程技师学院，支持湖北工程学院建设新型工程大学、湖北职业技术学院争创全国高职院校50强。

实施健康孝感计划。推动全民健身生活化，配套完善全民健身设施，市场化配建一批健身广场，鼓励机关和企事业单位非工作时间向社会开放体育场所。网格化布局县域医共体，加快现代医院管理制度建设，开展优质服务基层行活动。招录乡村全科助理医生200名。加快建设8个医疗卫生项目⑳。推进“医养结合”，实施“互联网+医疗健康”便民行动，加强职业病和重点疾病防控，适龄儿童疫苗接种率90%以上。

实施文化惠民计划。广泛开展“起点阅读”“朝读经典”“书香澴川·文化孝感”等全民阅读活动，配套完善一批村文化广场、基层文体中心，开放运营市文化中心，推进“金秋演出季”和农村公益电影放映，发展公益文化事业。推进长江国际文化广场建设，加快孝感麻糖米酒申遗，筹办孝文化旅游节、楚剧展演和新中国成立70周年系列文化活动，创编《孝·感天地》大型音乐剧，扶持文化产业，丰富文化生活。

（六）聚焦攻坚克难，深化职责“双履”。我们在履行发展主责的同时，要切实担起一岗双责的主体责任，打好打赢三大攻坚战，守土负责、守土尽责、齐抓共管、形成合力。

夺取精准脱贫新胜利。坚持现行标准，紧扣“准实”要求，下足绣花功夫，确保92847人脱贫、104个贫困村出列，大悟、孝昌2个贫困县摘帽，已脱贫人口持续稳定增收。强化产业扶贫支撑，“一户一策”稳固家庭经营，“一村一品”壮大村集体经济。兜牢健康扶贫底线，对23种大病贫困患者开展集中救治，家庭医生签约服务覆盖所有贫困人口。全年资助建档立卡贫困学生12万人次。推广“一自三合”[23]模式，扶贫小额信贷快办快放、应贷尽贷。开展社会保障兜底专项行动，农村低保量化提标。深化“四个遍访”[24]，用情结对帮扶，正面引导预期，激发脱贫原动力，提高扶贫效益和脱贫质量。

树立污染防治新标杆。全力推进长江大保护十大标志性战役，全面完成中省环保督察问题整改销号，节能降耗和“四个三”重大生态工程[25]完成省定目标任务，市高新区通过国家循环化改造示范试点终期验收。区域联防联控，持续开展臭氧污染、机动车尾气、扬尘污染专项整治， PM2.5平均浓度稳中有降，空气质量优良天数稳中有增。开展河（湖库）长制提档升级年行动，实行澴河流域横向生态补偿，“五水”共治[26]、水岸同治、流域联治，16条黑臭支流禁止污水直排，乡镇污水处理厂全部正常运营，30个市控断面三类以上水质达83%以上，8个国控断面水质稳定达标，饮用水源水质全部达标。建立土壤环境监测网络，“一控两减三基本”[27]治理农业面源污染，化肥农药再减5%，安陆、孝昌完成畜禽养殖废弃物资源化利用“整县推进”任务。建设“六类厕所”14.43万座。开展全域绿化，造林15万亩，通道绿化820公里、河网绿化260公里，创建省级森林城镇3个、绿色示范村150个，争创一批国家园林城镇、森林乡村、森林人家。高质量完成全国第四次经济普查和全省第五次森林调查工作。

筑牢风险防控新阵线。加强重点企业、非法集资、互联网金融等风险监测，市县乡三级联创金融信用单位，优化金融生态，创建平安金融。加强政府债务内控管理，依法合规举债，严控增量，消化存量。人防、物防、技防“三位一体”，社区、社工、社会组织“三社联动”，行政调解、人民调解、司法调解“三管齐下”，立体化信息化治安防控、扫黑除恶、打击犯罪“三措并举”，加强电诈、黄赌毒综合整治，提高社会治理和风险管理能力，提升“一感两度两率”；强化安全生产隐患排查、食品药品安全监管，加强应急队伍建设和演练，提高防灾减灾、救灾应急能力，坚决防范重大安全事故、重大群体事件发生，创建平安孝感。

（七）聚焦公平效率，深化政务“双提”。良好的政务环境是高质量发展的软实力。我们转观念、转职能，以信息化促规范化、公开化，实现政务服务提速提效。

优化政务服务。深化 “一网一门一次”[28]改革，方便群众网上办事，政务服务“一窗”分类受理事项达85%以上。加快统一身份认证、电子证照库和政务信息共享服务，实体大厅与网上办事深度融合，线上线下功能互补、合一通办。持续开展“三减”行动[29]，精简审批事项，优化再造流程，压缩办事时限，提高网办事项“三级八同”[30]标准质量，让更多高频事项“最多跑一次”。完善全市政务服务热线，加快对接湖北12345在线服务平台，让政策规定、公共服务贴近实际、贴近群众。

创新政务管理。推进“互联网+政务管理”，建设五大公共基础数据库，打造全市统一的数据交换共享平台、政务数据分析平台、智慧城市信息云平台，市政务云与省楚天云对接，提高政务管理信息化、标准化水平。优化“五化”管理平台，重大事项全入库、严出库；以过程管控确保目标落实，以节点管理倒逼责任担当，以成事之道促进合力形成。加强信用体系建设与运用，持续开展市场秩序整治，广泛开展“双随机一公开”，强化事中事后监管，规范市场秩序。

拓展政务公开。畅通沟通渠道，完善公众参与机制。严格执行政府信息公开条例，公开为常态、不公开为例外，全面推进行政决策公开、执行公开、管理公开、服务公开和结果公开。加强政府门户网站、“两微一端”平台建设，推进政府网站集约化，政民互动、回应关切，提升群众满意度、政府公信力。

（八）聚焦人民满意，深化作风“双严”。政府自身建设是高质量发展的根本保证。我们要把人民满意作为不懈追求，从严管理、从严要求，提能力、强素质，努力建设人民满意政府。

突出政治标准。旗帜鲜明讲政治，树牢“四个意识”，坚定“四个自信”，践行“两个维护”，落实“十个带头”[31]，提高政府工作的政治站位。坚决贯彻落实上级党委政府决策部署，坚持党的领导，当好“行动政府”。树牢团队意识，正确处理分工与合作、主角与配角、分管与统筹的关系，团结协作、互相配合、同舟共济。

注重依法行政。执行《孝感市法治政府建设实施方案》，把依法行政贯穿工作始终，依法决策、履职、担责，建设法治政府。自觉接受人大、政协和人民群众监督，搭建人大议案、建议和政协提案信息化平台，提高办理质效。广泛听取民主党派、工商联、无党派人士和人民团体意见，支持工会、共青团、妇联、科协、侨联等群团组织发挥更大作用，汇集民智、接受监督。深入开展“七五”普法，推行行政综合执法体制改革，加强行政复议规范化建设，严格规范公正文明执法。

倡导“两干”[32]作风。把执行、落实作为政府价值所在。紧扣价值、问题、规律导向和政策、事实底线谋事，用心用情用力干事，坚持“功成不必在我、建功必须有我”尽事，大力倡导“马上办、我来办、连夜办”，持续整治“门好进、脸好看、事难办”和“为了不出事、宁可少干事”等问题，精减无谓事务、无用程序，根治“留痕”思维，不让工作在此耽误，不让差错在此发生，不让群众在此冷落，不让形象在此受损。法定职责、议定事项、既定目标，一抓到底、勤督实查、严评实考，提高效率！

建设清廉政府。认真落实全面从严治党主体责任，加强政府系统廉政建设，严守纪律规矩，规范和加强资金管理、工程招投标、国有资产管理，强化审计监督，扎牢织密制度笼子，用制度管人管钱管事。坚决落实中央八项规定精神，持续整治形式主义、官僚主义，旗帜鲜明反对腐败，全力支持纪委监委工作，无禁区、全覆盖、零容忍，严肃查处不正之风和违纪违法案件。严管厚爱并重、激励约束并举，着力打造“忠诚、干净、担当”的公务员队伍。

各位代表，征程万里风正劲，扬帆起航争朝夕。让我们紧密团结在以习近平同志为核心的党中央周围,在省委、省政府和市委坚强领导下，不忘初心、牢记使命，与时代同行、与人民同心，在实现孝感高质量发展的奋斗征程中，开创新局面、干出新业绩、建功新时代！
